# When I say … neurodiversity paradigm

**DOI:** 10.1111/medu.15565

**Published:** 2024-11-19

**Authors:** Sebastian Charles Keith Shaw, Megan E. L. Brown, Neera R. Jain, Riya Elizabeth George, Sarah Bernard, Megan Godfrey‐Harris, Mary Doherty

**Affiliations:** ^1^ Department of Medical Education Brighton and Sussex Medical School Brighton and Hove UK; ^2^ School of Medicine Newcastle University Newcastle UK; ^3^ Centre for Medical and Health Sciences Education, Faculty of Medical and Health Sciences University of Auckland Auckland New Zealand; ^4^ MBA in Global Management, School of Management and Finance University College London London UK; ^5^ Faculty of Medicine and Dentistry Queen Mary University of London London UK; ^6^ Department of Geriatric Acute and Rehabilitation Medicine Sir Charles Gairdner Osborne Park Healthcare Group Perth Western Australia Australia; ^7^ Darent Valley Hospital Dartford and Gravesham NHS Trust Dartford UK; ^8^ UCD School of Medicine University College Dublin Dublin Ireland; ^9^ Department of Clinical Neuroscience Brighton and Sussex Medical School Brighton UK

## Abstract

In this “When I say…” article, the authors argue for a philosophical transformation in how medical education values different ways of thinking and learning: the neurodiversity paradigm.

New paradigms require new language. The term ‘neurodiversity’ is often misunderstood and used interchangeably with neurodivergence. Instead, neurodiversity describes the infinite, natural variation of neurocognitive functioning in human minds.^1^ ‘Neurodiverse’ is, therefore, a societal descriptor, outlining this variation across the population. By contrast, ‘neurodivergent’ refers to those who differ from societally perceived norms of brain or mind function—or from the majority, who are sometimes described as ‘neurotypical’. Neurodiversity should not be thought of as a single or even static concept. This raises the question what do people mean when they say ‘neurodiversity’? On the one hand, we might consider the biological fact that all human brains differ to varying degrees. This is not, however, what we are referring to. Next, we might consider the neurodiversity *movement*, grounded in the disability rights movement—the on the ground advocacy driving forwards justice for neurodivergent people culturally, socially and politically.^1^ This may indeed be what some refer to when discussing ‘neurodiversity’ as an undifferentiated term. Here, however, we shall discuss the neurodiversity *paradigm*. This refers to the conceptual beliefs, or worldview, underpinning the assertion that a neurodiverse society is made up of neurotypical and neurodivergent people—and that being neurodivergent is no less worthy than being neurotypical.

Diversity of thought, perspective and experience adds to the rich nuance required to understand the complexity of medical practice and education. A diverse medical workforce also benefits the general population. For example, neurodivergent doctors may help to alleviate barriers to healthcare access for neurodivergent patients.^2^ Medical education is, however, failing neurodivergent learners, who continue to face significant challenges, despite efforts to promote student support and accessibility.^3^, ^4^ Why are our current approaches insufficient, and what needs to change? In this ‘When I say …’ article, we leverage our unique lived experiences to offer a diversity of perspectives on current understandings in this area. We argue that medical education will fundamentally fail in its mission to support neurodivergent learners unless we undergo a significant transformation in how we value different ways of thinking and learning. We conceptualise this philosophical transformation as a shift towards the neurodiversity paradigm.

First and foremost, the neurodiversity paradigm concerns itself with social justice. Drawing on the concept of power, this paradigm promotes critical thinking around things we have previously passively accepted as real. This considers the influence of power on how we constructed such historical ‘truths’ and how these are then maintained as part of the wider status quo. Prior to this, traditional understandings of neurodivergence were created and maintained by the neurotypical majority. This led to external perspectives of people being disordered. For example, Autistic people were previously—and erroneously—thought to feel no empathy because they displayed empathy in ways that fell outside of normative societal expectations, which went unrecognised by medical professionals and researchers. This led to a ‘fallacy of misplaced concreteness’, where a perspective about the way neurodivergent people are, developed through decades of research from a deficit perspective, was mistaken for reality and continually reinforced. In that sense, it may be easy to ‘prove’ something if we do not reflect critically on its philosophical underpinnings. The neurodiversity paradigm brings a critical perspective to this area.

Paradigms are sets of ideas through which people engage with reality and form knowledge, which are determined by our ontological and epistemological beliefs.^5^ Ontology considers the nature of reality—what something *is*. This might consider one objective truth (‘realism’, where we believe there is a single reality that is separate to those experiencing or perceiving it) or that reality is more subjective (‘relativism’, where reality may instead be different for different people). For example, if two people attend an event and experience it very differently, is one of them correct and the other wrong, or might the reality be dependent on the person perceiving it? Epistemology then considers how we create knowledge, entwined with our ontological beliefs.^5^ For example, might the best approach be objective and free of bias, or subjective and embracing human experience? Digging deeper, this could also consider our aims in making knowledge, such as seeking to improve human existence or promoting social justice. In that sense, it asks *what* knowledge is meaningful and *how* we can best create it.

Ontologically, the neurodiversity paradigm argues that neurodivergent people are *different*—not flawed, impaired, or disordered. Difference does not entail inherent lack of worth, regardless of whether difference is personally experienced as an impairment, a disability, or an advantage. Societal factors can contextually *disable* neurodivergent people due to a mismatch between their differences and any given expectations. Such factors might be historical, political, cultural, social or environmental in nature. This is different from a strengths‐based perspective, which promotes a focus on celebrating *strengths*. This accidental conflation of these has attracted criticism over the years, with people thinking the neurodiversity paradigm did not account for disablement or that it asserted that neurodivergent people had inherent ‘superpowers’. Instead, this paradigm argues that strengths and weaknesses are both contextual, depending on the aforementioned mismatch between our differences and the given situation, systems, structures and expectations.

Epistemologically, the neurodiversity paradigm argues that neurodivergent people should be central to the creation of knowledge around neurodivergence. Research should be grounded in neurodivergent community perspectives and should seek to advance social justice. In essence, the minority‐group status of neurodivergent people led to them having no meaningful voice in the academic world for many years. This was an example of an ‘epistemic injustice’—an injustice in the way we have created knowledge, which has systematically led to misunderstanding and one‐sided perspectives.^3^ The lack of neurodivergent voices shaping our discourse has led to flawed practices. One such example is policies to screen for neurodivergence following exam failures. Such policies remain commonplace in both undergraduate and postgraduate medical education in the UK. While well intended, this practice draws on a deficit‐based view of neurodivergence and reinforces associations with failure and thus promotes on‐going stigma. Small changes, such as offering optional screening to all students on admission to their courses, could help to break the association with failure in this example.

The neurodiversity paradigm calls for a shift in how we conceive of medical education and suggests that neurodivergent people must be involved in decision‐making about how we move forward. There are many ways we can learn from and embody this paradigm. Given the scope of this paper, we have identified a few high priority applications to consider.

- Active consideration of discrimination and ableism. How have our systems, policies and practices assumed a certain kind of body and mind in our conception of physicians? This is necessary to shift systemic and institutional processes that block neurodivergent people from thriving.
- Meaningfully involving neurodivergent people in our research, curriculum design and development. This is an active step to tackle epistemic injustice and build towards inclusion and universal design. Engaging authentically with neurodivergent people can help identify additional applications of the paradigm within medical education.
- Seek local, diverse neurodivergent perspectives. The contexts that disable neurodivergent people vary across cultures and environments worldwide. Neurodivergent people at the intersections of marginalisation encounter education and medical practice in ways that are shaped by local social histories and the legislative, policy and governance frameworks embedded in their institutions.

Reflecting upon our own prevailing assumptions is key to mobilising this paradigm shift. We encourage readers to reflect upon and interrogate the following assumptive statements. Do these resonate with your personal experiences? What challenges might these raise if left unquestioned? What perspectives might you need to incorporate to shift your understanding?

- ‘But you don't *seem* Autistic.’
- ‘Isn't everyone a little bit neurodiverse?’
- ‘All doctors *need* to be able to …’

## AUTHOR CONTRIBUTIONS

**Sebastian Charles Keith Shaw:** Conceptualization; writing—original draft; writing—review and editing. **Megan E. L. Brown:** Conceptualization; writing—review and editing. **Neera R. Jain:** Conceptualization; writing—review and editing. **Riya Elizabeth George:** Conceptualization; writing—review and editing. **Sarah Bernard:** Conceptualization; writing—review and editing. **Megan Godfrey‐Harris:** Conceptualization; writing—review and editing. **Mary Doherty:** Conceptualization; writing—review and editing.

## Data Availability

Data sharing not applicable to this article as no datasets were generated or analysed during the current study.
